# A tailored approach to the management of post-haemorrhagic hydrocephalus

**DOI:** 10.1007/s00381-023-06214-6

**Published:** 2023-11-12

**Authors:** Benjamin J. Hall, John C. Duddy, Katerina Apostolopoulou, Benedetta Pettorini

**Affiliations:** 1 Department of Neurosurgery, Alder Hey Children’s Hospital NHS Foundation Trust, Liverpool, UK; 2 Department of Neurosurgery, The Walton Centre NHS Foundation Trust, Liverpool, UK; 3 Institute of Infection, Veterinary and Ecological Sciences (IVES), The University of Liverpool, Liverpool, UK

**Keywords:** Neuro-endoscopy, Lavage, Intraventricular haemorrhage, Hydrocephalus

## Abstract

**Purpose:**

Neuro-endoscopic lavage (NEL) is an increasingly popular intervention for intraventricular haemorrhage (IVH) and post-haemorrhagic hydrocephalus (PHH), with considerable variation in technique dependent on clinician and clinical circumstances. Whilst efforts to standardise the technique are ongoing, this work describes a tertiary centre experience utilising NEL, highlighting potential caveats to standardisation.

**Methods:**

A retrospective review of electronic case notes for patients undergoing temporising surgical intervention for IVH between 2012 and 2021 at our centre was performed. Data collected included (i) gestational age, (ii) aetiology of hydrocephalus, (iii) age at time of intervention, (iv) intervention performed, (v) need for permanent CSF diversion, (vi) ‘surgical burden’, i.e. number of procedures following primary intervention, and (vii) wound failure and infection rate. Data was handled in Microsoft Excel and statistical analysis SPSS v27.0

**Results:**

49 neonates (*n* = 25 males) were included. Overall mean gestational age was 27 weeks and at intervention 35 + 3 weeks. IVH was the predominant cause of hydrocephalus (93.8%) and primary surgical interventions included insertion of a ventriculosubgaleal shunt (VSGS) in *n* = 41 (83.6%) patients, NEL in *n* = 6 (12.2%) patients and insertion of an EVD in *n* = 2 (4.1%). *N* = 9 (18.4%) patients underwent NEL at some point during the time interval reviewed; n = 4 (8.2%) received NEL monotherapy and *n* = 5 (10.2%) also received a VSGS. Rate of conversion to definitive CSF diversion between NEL (*n* = 8, 88.9%) and VSGS cohorts (*n* = 37, 92.5%) was not significantly different (*p* = 0.57), nor between NEL alone (*n* = 3, 75%) and NEL + VSGS (*n* = 5, 100%) (*p* = 0.44). None of the patients that underwent NEL monotherapy had any wound issues or CNS infection as a result of the initial intervention, compared to *n* = 3 (60%) of those that underwent NEL and implantation of VSGS (*p* = 0.1).

**Conclusion:**

Both NEL and VSGS are effective in temporising hydrocephalus in neonates, occasionally offering a definitive solution in and of themselves. The benefit of dual therapy however remains to be seen, with the addition of VSGS potentially increasing the risk of wound failure in an already vulnerable cohort.

## Introduction

Neonatal intraventricular haemorrhage (IVH) and subsequent post-haemorrhagic hydrocephalus (PHH) are prevalent and devastating sequalae of premature birth and low birth weight [1]. With ever advancing neonatal care, survival of extreme prematurity is becoming more commonplace and, with it, the associated morbidity. The optimal management approach to IVH and PHH remains a hotly debated topic, with permanent CSF diversion through ventricular shunts remaining the mainstay of long-term treatment. However, the low birth weight and complex comorbidities associated with prematurity result in an expectedly high failure rate of shunts in this infant cohort. Such high failure rates, as well as the neurodevelopmental impact of IVH independent of PHH, have led to work towards alternative interventions in the perinatal period.

The DRIFT trial popularised the concept of fibrinolytic therapy in reducing clot burden [2], whilst the role of ventriculosubgaleal shunts (VSGS), endoscopic third ventriculostomy (ETV), and ventricular access devices (VADs) provides a range of temporising measures in PHH until shunting becomes feasible. Neuro-endoscopic lavage (NEL) has grown in popularity in recent years, demonstrating a comparatively greater safety profile than DRIFT in relation to risk of haemorrhage, whilst resulting in similar outcomes in terms of shunting, cognitive function, and mortality as alternative treatment modalities [3]. Notable variation in technique and an absence of randomised trial data have culminated in efforts to standardise the approach to NEL in the UK [4]. Whilst standardisation of care often remains the goal across healthcare, the IVH patient cohort demonstrates a substantial degree of heterogeneity that may preclude a standard approach when considering NEL.

This study presents a UK tertiary centre experience utilising NEL over the last decade, highlighting the need for further comparison between temporisation techniques in PHH before a standardised approach can be adopted fully.

## Methods

A retrospective review of all neonates admitted to our paediatric neurosurgical department between 2012 and 2021 requiring temporisation of hydrocephalus was performed. All modalities of CSF diversion were reviewed, including VSGS, NEL, EVD (external ventricular drain), Ommaya reservoir and any combination of these. Electronic case notes were reviewed for patient details including (i) gestational age at birth, (ii) birth weight, (iii) aetiology of hydrocephalus, (iv) grade of IVH, and (v) surgical intervention. Outcome data collection included (i) need for definitive CSF diversion, (ii) infection and wound failure rates, (iii) valve choice, (iv) overall shunt survival, (v) ‘surgical burden’: defined as the number of hydrocephalus-related operations undertaken during follow-up, (vi) development of and treatment required for loculated hydrocephalus, and (vii) mortality. Patients were grouped and analysed according to whether they underwent NEL, VSGS implantation, or both.

Data handling was performed in Microsoft Excel and statistical analysis in SPSS v27.0. Overall shunt survival is illustrated using Kaplan–Meier curves with log-rank testing to assess for statistically significant differences. Comparison of dichotomous variables is presented descriptively and compared with a chi-squared test, whilst ordinal data was assessed using the Mann–Whitney *U* test. Ranges (*R*), 95% confidence intervals (95% CI), and standard deviations (SD) are reported where appropriate, and statistical significance threshold was set at *p* < 0.05.

### Procedural technique

In our centre, a protocolised approach is taken towards managing post-haemorrhagic hydrocephalus. Initially, each patient undergoes an MRI brain in order to (i) more accurately assess intraventricular blood load and the presence of organised clots and (ii) delineate the presence of any third ventricular obstruction or intraventricular membranes. Following formal radiological report and neurosurgical review of the MRI, the choice of temporisation technique is then decided. In circumstances wherein blood load is minimal and accompanied by few organised clots, a solitary ventriculosubgaleal shunt is inserted under image guidance without endoscopic intervention. In those whom blood load is greater or there is a substantial number of organised clots, endoscopic washout is employed. Decision to also implant a VSGS as an adjunct to NEL is determined on a case-by-case basis. All procedures are performed in theatre, under general anaesthetic and with use of antibiotic prophylaxis. Skin incision is made with undermining of a subgaleal pocket for VSGS (if indicated), before formation of a burr hole and insertion of the endoscope into the frontal horn of the ventricle. Clots are gently dislodged and blood washed out. Further endoscopic fenestration in the form of pellucidotomy or ETV is also employed on an individualised basis, typically in circumstances where there is evidence of isolated ventriculomegaly or intraventricular membranes.

## Results

*N* = 57 neonates were identified during the time interval as having undergone temporising CSF diversion. *N* = 8 patients were excluded from analysis: *n* = 4 developed spontaneous CNS infection prior to any surgery, *n* = 3 died of complications related to prematurity in the neonatal period prior to definitive shunt insertion, and *n* = 1 moved out of area with VSGS in situ. *N* = 49 patients were therefore included for follow-up analysis, *n* = 25 (51.0%) of whom were male.

Overall mean gestational age at birth was 28.1 weeks (*R*, 17–40; SD, 4.8): 28 weeks (SD: 4.5) for NEL as primary procedure and 28 weeks (SD, 4.8) for VSGS (*p* = 0.5). Overall mean age at primary intervention was 6.4 weeks (SD, 5.5): 10.7 weeks (SD, 13.2) for those undergoing NEL and 6.0 weeks (SD, 3.4) in those receiving VSGS (*p* < 0.01). Overall mean birth weight was 2.0 kg (*R*, 0.8–3.6; SD, 0.8); for NEL patients, it was 1.6 kg (SD, 1.5) and for VSGS 1.4 kg (SD: 1.3) (*p* = 0.8). IVH was the underlying aetiology in *n* = 46 (93.8%) patients, an antenatal diagnosis of hydrocephalus (*not otherwise specified*; NOS) was found in *n* = 2 (4.1%) patients, and haemorrhage secondary to a Vein of Galen malformation was responsible in *n* = 1 (2.0%) patient. Of those diagnosed with IVH, *n* = 36 (78.2%) were Grade IV, *n* = 8 (17.4%) were Grade III, and *n* = 2 (4.3%) were Grade II.

The primary intervention for temporary CSF diversion was VSGS in *n* = 41 (83.6%) patients, NEL in *n* = 6 (12.2%) patients, and insertion of an EVD in *n* = 2 (4.1%) patients. *N* = 9 (18.4%) patients underwent NEL at some point during the time interval reviewed; *n* = 4 (8.2%) received NEL monotherapy, and *n* = 5 (10.2%) also received a VSGS. Pellucidotomy or cyst fenestration was performed simultaneously in *n* = 2 (22.2%) cases, and an ETV was performed in *n* = 1 (11.1%). case. Details of all patients undergoing NEL, either as monotherapy or in conjunction with additional CSF diversion, are outlined in Table 1. *N* = 2 patients that underwent implantation of a VSGS as monotherapy unfortunately experienced blockage of the shunt and therefore returned to theatre. At this point, both then underwent NEL and re-implantation of a new VSGS thereafter.

**Table 1:** All patients undergoing neuro-endoscopic lavage (NEL), either as monotherapy or in conjunction with additional CSF diversion

**Patient**	**Grade**	**1st surgery**	**2nd surgery**	**NEL details**	**Complications?**	**Surgeries**	**Procedures**
1	IVH 2	NEL + ETV	VP shunt	ETV + NEL; most clots removed	Wound failure; multiple early shunt revisions	12	1 ETV/NEL, 9 shunts, 2 endo
2	IVH 4	NEL	VP shunt	Details missing	Nil	6	1 NEL, 4 shunts, 1 endo
3	IVH 4	NEL (× 2)	VSGS	Details missing	Nil	6	2 NEL, 1 VSGS, 3 shunts
4	IVH 3	NEL	VP shunt	Most clots removed, unilateral	Nil	2	1 NEL, 1 shunt
5	IVH 4	EVD	NEL + VSGS	Most clots removed, unilateral	Wound leak with VSGS in situ; not infected	3	1 EVD, 1 NEL/VSGS, 1 shunt
6	IVH 4	NEL + VSGS	VP shunt	All clots cleared; pellucidotomy + BL NEL	Nil	2	1 NEL, 1 shunt
7	IVH 4	NEL	Nil required	BL IVH R > L so unilateral NEL, some clots removed	Nil	1	1 NEL
8	IVH 4	VSGS	NEL + new VSGS	NEL + endoscopically fenestrated cyst	1st VSGS infected	5	1 VSGS, 1 NEL/VSGS, 1 Ommaya, 1 endo, 1 shunt
9	IVH 4	VSGS	NEL + new VSGS	Most clots removed, unilateral	1st VSGS blocked	10	2 VSGS, 8 shunt

*VSGS* ventriculosubgaleal shunt, *EVD* external ventricular drain, *ETV* endoscopic third ventriculostomy, *VP* ventriculoperitoneal, *BL* bilateral

Overall median follow-up was 286 weeks, and definitive CSF diversion was required in *n* = 45 (91.8%) patients during this time. There was no significant difference in the proportion of patients requiring definitive CSF diversion between NEL (*n* = 8, 88.9%) and VSGS cohorts (*n* = 37, 92.5%) respectively (*p* = 0.57), nor between NEL alone (*n* = 3, 75%) and NEL + VSGS (*n* = 5, 100%) (*p* = 0.44). Definitive CSF diversion took the form of ventriculoperitoneal (VP) shunts in *n* = 39 (79.6%) patients and ventriculoatrial (VA) shunts in *n* = 6 (12.2%), whilst the choice of the first valve varied between programmable valves in *n* = 31 (63.2%) patients and fixed pressure valves in *n* = 14 (28.6%).

Overall mean time to the first shunt (TTFS) was 5.2 weeks (range, 0.6–12.9; SD, 2.9); in the NEL group median TTFS was 2.9 weeks (SD: 1.9) compared to 5.8 weeks (SD, 2.8) in the VSGS group (*p* = 0.01). The average number of future procedures did not differ significantly between intervention groups, with a mean of *n* = 5.2 (SD, 3.8) for the NEL cohort, compared to *n* = 5.1 (*r*, 3.3) in the VSGS cohort (*p* = 0.9).

**Fig. 1:**
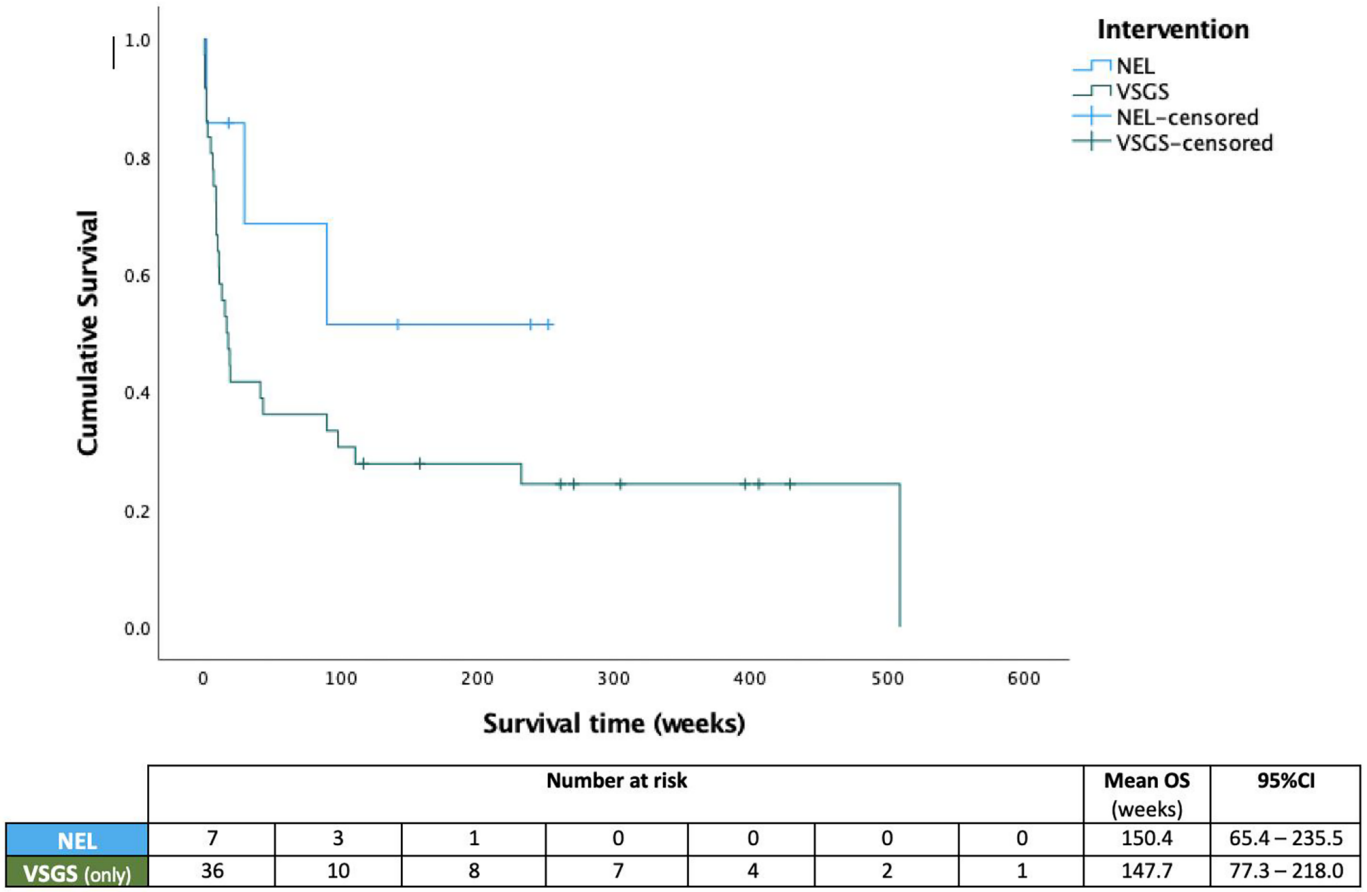
Cumulative survival of the first ventricular shunts implanted in patients undergoing NEL as monotherapy or in conjunction with VSGS (NEL) or VSGS implantation alone (VSGS)

**Fig. 2:**
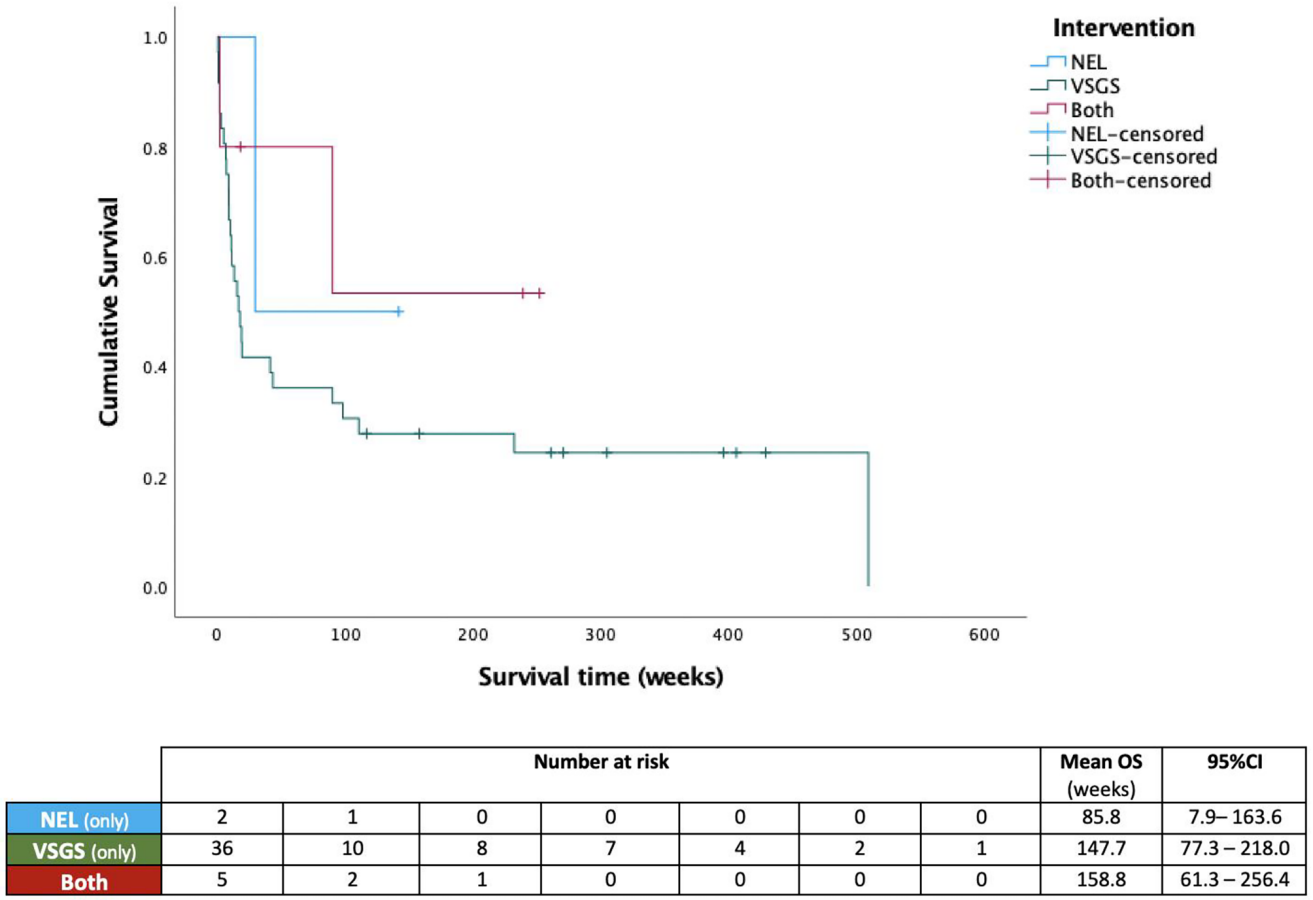
Cumulative survival of de novo ventricular shunts implanted in patients undergoing NEL as monotherapy, VSGS implantation alone (VSGS), or a combination of the two (both)

Comparing de novo shunt survival between groups, mean overall survival was 150.4 (95% CI, 65.4–235.5) weeks for patients undergoing NEL at any point, compared to 147.7 (95% CI, 77.3–218.0) with VSGS alone (*p* = 0.16) (Fig. 1). De novo shunt survival compared between each subgroup was 85.8 (95% CI, 7.9–163.6) weeks after NEL monotherapy and 158.8 (95% CI, 61.3–256.4) with simultaneous NEL and VSGS implantation (*p* = 0.3) (Fig. 2). Wound failure or CSF leak occurred in *n* = 13 (26.5%) patients, all of whom underwent VSGS insertion either as monotherapy or in conjunction with NEL, equating to 28.9% of VSGS cases. Of those facing wound issues, *n* = 5 (38.5%) developed CNS infection as a result of the CSF leak. No patients that underwent NEL monotherapy had any wound issues or CNS infection as a result of the initial intervention, compared to *n* = 3 (60%) of those that underwent NEL and implantation of VSGS (*p* = 0.1). Those who experienced wound issues were found to be significantly younger, with a mean age of 4.5 weeks (SD: 2.3), compared to 7.2 weeks (SD, 6.3) in those who did not (*p* = 0.05), though neither weight or gestational age at birth were significantly associated with risk of wound failure or infection (*p* > 0.1). Median number of operations related to hydrocephalus, or ‘surgical burden’ was *n* = 5 per patient in both treatment groups (*r*, 1–15) (*p* = 0.9).

Multiloculated hydrocephalus, either in the form of supratentorial intraventricular cysts or a ‘trapped’ fourth ventricle, developed in *n* = 5 (55.6%) NEL patients and *n* = 18 (45%) VSGS patients (*p* = 0.22). Endoscopic intervention, either through cyst fenestration or aqueductoplasty, was indicated in *n* = 4 (44.4%) NEL patients and in *n* = 11 (27.5%) VSGS patients (*p* = 0.41). Of the patients who required endoscopic intervention for loculated hydrocephalus, *n* = 9 (60.0%%) required one endoscopic procedure, *n* = 4 (26.7%) required two endoscopic procedures and *n* = 2 (13.3%) required three endoscopic procedures.

## Discussion

IVH and PHH are frequently observed in the premature population and are associated with particularly low birth weight and gestational age [5]: variables that are both independently also associated with poorer shunt survival. Thus, ensuring the earliest interventions are appropriately selected is crucial. This data highlights the clinical heterogeneity in this patient cohort, as well as the variability in choice of intervention [6]. Whilst a range of interventions was employed, NEL and VSGS were the most common, in either isolation or concomitantly.

Severity of IVH is well recognised as being associated with more severe neurodevelopmental delay, as well as risk of PHH [7]. Whilst initial brain injury secondary to IVH is expected to contribute to poorer neurodevelopmental outcomes, the development of hydrocephalus and its ongoing management also plays a substantial role. 78.2% of this cohort were referred following Grade 4 IVH, putting them at a particularly high risk of requiring definitive CSF diversion. Neither NEL or VSGS were significantly more likely to reduce the need for definitive shunting (*p* = 0.57), though patients undergoing NEL underwent their definitive shunt insertion after a significantly shorter interval of 2.9 weeks (SD, 1.9) compared to 7.1 weeks (SD, 8.3) in the VSGS group (*p* = 0.01). Reasons for this discrepancy may be multiple, the most obvious being that VSGS provide continuous CSF drainage until conversion at a later date. It is also standard practice at our centre to avoid implantation of any permanent shunt device until the patient is at least 2 kg in weight; the mean weight of those undergoing NEL was appreciably, though non-significantly, higher than that of the VSGS cohort (2.3 kg vs 1.8 kg), perhaps also contributing to the delay in shunt implantation after VSGS.

However, whilst the continuous CSF diversion provided by VSGS enables an opportunity for the patient to be clinically optimised for shunt insertion, this longer interval is not without risk. The prevalence of wound failure or CSF leak was noticeably higher in the VSGS cohort in this series at 33.3%, compared to 0% in those undergoing NEL alone. Within the VSGS group, a third of those who developed wound issues ultimately also developed CNS infections; resulting in prolonged admissions and further risk to neurodevelopment and life. Whilst the sample size was too small to determine whether this was a statistically significant difference, the discrepancy is striking and may provide further credence to the role of NEL monotherapy in managing this cohort of particularly vulnerable patients. Of note, those who suffered wound failure were statistically significantly younger than those who did not, highlighting the fragility of skin healing in this cohort and perhaps a role for improved patient selection when determining which intervention to utilise.

Whether choice of temporising measure has a significant impact on the survival of de novo shunts remains to be seen. Whilst shunt survival varied between NEL patients with and without implantation of VSGS, the small sample sizes in these groups prohibit interpreting whether a true difference exists, as inevitably there was no statistical significance identified (*p* = 0.9). Should prolonged de novo shunt survival be confirmed in a larger cohort, then this would provide additional support for the notion of including VSGS implantation as standard following NEL. Further work into the mechanistic explanation for such an effect must also be undertaken: whether it be due to a difference in the degree of intraventricular debris cleared, or perhaps as a result of a longer interval between temporization and definitive shunt implantation.

Multiloculated hydrocephalus is a challenging clinical entity, often seen within this cohort of premature infants with IVH or meningitis. The exact pathophysiology underlying the development of intraventricular membranes is as yet undefined but is felt to be related to an inflammatory reaction, either related to blood, infection, or chronic shunting [8]. Inevitably, the presence of multiple intraventricular membranes increases the risk of shunt failure, but also the requirement for endoscopic intervention and yet more surgical burden. Whilst the prevalence of multiloculated hydrocephalus was not significantly different between the NEL and VSGS groups (55.6% vs 35%, *p* = 0.22) in this series, the variation highlights an area that may warrant further investigation in larger, future cohorts.

At our centre, the typical criteria for each procedure involve VSGS insertion alone in those with minimal clot burden, NEL alone in those with substantial clot burden, and the utilisation of both NEL and VSGS is decided on a case-by-case basis. The heterogeneity seen not only in the clinical state of the patient but also the blood distribution and presence of clots results in procedural variation between cases (Table 1). With regard to intraventricular technique itself, the primary goal should remain as maximal safe lavage of any clots amenable to removal, without undue manipulation or prolongation of the procedure such that the patient is put at greater risk of harm. In these circumstances whereby clots are particularly challenging to remove, the addition of a VSGS after NEL may be appropriate. Of note, on reviewing those patients (*n* = 2) that initially received a VSGS before then returning to theatre for NEL and VSGS revision due to shunt blockage: both were patients admitted earlier in the series. Had these patients been treated during our more contemporary practice, they would more likely have undergone NEL as primary intervention based on their initial imaging.

Whilst the addition of VSGS evidently delays time until definitive shunt implantation, though whether this outweighs the associated risk of wound failure remains to be seen and requires comparison between larger cohorts. Contrary to current attempts to standardise NEL, the discrepancy in wound issues seen in this group suggests mandatory implantation of a VSGS following NEL may not be necessary, given the risks associated and no greater chance of providing shunt independency.

### Limitations

As with many studies examining the role of NEL, the sample size and retrospective nature of the data are an inherent limitation. Discrepancies between treatment modalities in areas such as wound failure and infection rates would be better described by a larger sample size, and prospectively collected data would enable a more thorough and detailed description of the operative techniques utilised.

## Conclusion

Both NEL and VSGS are effective in temporising hydrocephalus in neonates, occasionally offering a definitive solution in and of themselves. The benefit of dual therapy however remains to be seen, with the addition of VSGS potentially increasing the risk of wound failure in an already vulnerable cohort. Whilst standardisation remains a goal in temporising PHH, the concept requires further work, with greater emphasis made towards improving patient selection, as well as discerning the benefit from each intervention individually.

## Data Availability

The datasets generated and/or analysed during the current study are available from the corresponding author on reasonable request.
